# Racemic tricarbon­yl[7-meth­oxy-2-(η^6^-phen­yl)chromane]­chromium(0)

**DOI:** 10.1107/S1600536811008683

**Published:** 2011-03-12

**Authors:** Mukut Gohain, Theunis J. Muller, Barend C. B. Bezuidenhoudt

**Affiliations:** a Department of Chemistry, University of the Free State, PO Box 339, Bloemfontein 9300, South Africa

## Abstract

In the title compound, [Cr(C_16_H_16_O_2_)(CO)_3_], the Cr^0^ atom of the Cr(CO)_3_ unit is coordinated to the phenyl ring of the flavan ligand in an η^6^ mode, with a normal arene-to-metal distance. The Cr(CO)_3_ unit exhibits a three-legged piano-stool conformation, while the dihydro­pyran ring displays a distorted envelope configuration. The phenyl ring is twisted away from the fused ring system by 25.5 (2)°. The meth­oxy group is almost coplanar with the phenyl ring [C_Me_—O—C_ar_—C_ar_ torsion angle = 8.46 (2)°]. The crystal packing is stabilized by inter­molecular C—H⋯O inter­actions.

## Related literature

For similar structures, see: van Tonder *et al.* (2010*a* ▶,*b* ▶) and for other related structures, see: van Tonder *et al.* (2009*a* ▶,*b* ▶). For the synthesis of the title compound, see: Müller *et al.* (1999 ▶) and for the sythesis of 7-meth­oxy­flavan-4-one, see: Sato *et al.* (2006 ▶). For standard bond lengths, see: Allen *et al.* (1987 ▶). For the importance of flavonoids in biological investigations, see: Rice-Evans & Packer (2003 ▶). For the use of tricarbon­yl(arene)chromium complexes in regioselective organic synthesis, see: Muschalek *et al.* (2007 ▶).
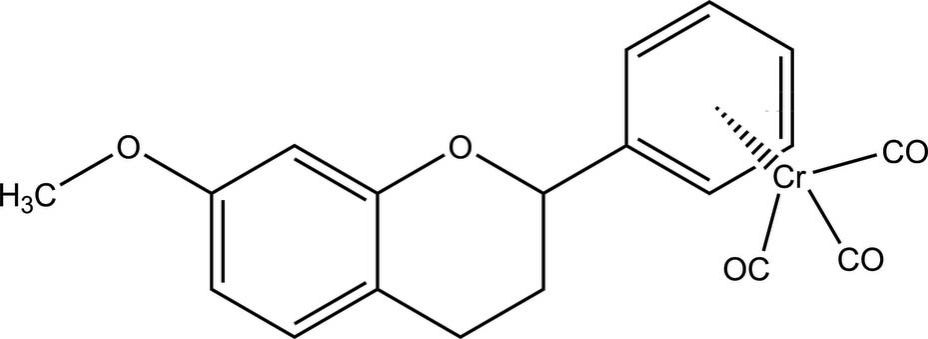


## Experimental

### 

#### Crystal data

[Cr(C_16_H_16_O_2_)(CO)_3_]
*M* *_r_* = 376.32
Monoclinic, 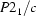

*a* = 9.7703 (5) Å
*b* = 19.1820 (9) Å
*c* = 8.8049 (4) Å
β = 97.494 (2)°
*V* = 1636.07 (14) Å^3^
*Z* = 4
Mo *K*α radiation
μ = 0.73 mm^−1^
*T* = 100 K
0.34 × 0.23 × 0.08 mm

#### Data collection

Bruker X8 APEXII 4K KappaCCD diffractometer
Absorption correction: multi-scan (*SADABS*; Bruker, 2008 ▶) *T*_min_ = 0.818, *T*_max_ = 0.942
29057 measured reflections
4069 independent reflections
3526 reflections with *I* > 2σ(*I*)
*R*_int_ = 0.033

#### Refinement

*R*[*F* ^2^ > 2σ(*F* ^2^)] = 0.034
*wR*(*F* ^2^) = 0.090
*S* = 1.00
4069 reflections
222 parameters
H-atom parameters constrained
Δρ_max_ = 0.73 e Å^−3^
Δρ_min_ = −0.52 e Å^−3^

### 

Data collection: *APEX2* (Bruker, 2008 ▶); cell refinement: *SAINT-Plus* (Bruker, 2008 ▶); data reduction: *SAINT-Plus*; program(s) used to solve structure: *SHELXS97* (Sheldrick, 2008 ▶); program(s) used to refine structure: *SHELXL97* (Sheldrick, 2008 ▶); molecular graphics: *DIAMOND* (Brandenberg & Putz, 2005 ▶); software used to prepare material for publication: *WinGX* (Farrugia, 1999 ▶).

## Supplementary Material

Crystal structure: contains datablocks global, I. DOI: 10.1107/S1600536811008683/hp2002sup1.cif

Structure factors: contains datablocks I. DOI: 10.1107/S1600536811008683/hp2002Isup2.hkl

Additional supplementary materials:  crystallographic information; 3D view; checkCIF report

## Figures and Tables

**Table 1:** Hydrogen-bond geometry (Å, °)

*D*—H⋯*A*	*D*—H	H⋯*A*	*D*⋯*A*	*D*—H⋯*A*
C4′—H4′⋯O2^i^	0.93	2.54	3.459 (2)	169
C2′—H2′⋯O4^ii^	0.93	2.46	3.153 (2)	132
C1—H1*C*⋯O4^iii^	0.96	2.57	3.314 (2)	134

Symmetry codes: (i) 
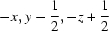
; (ii) 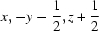; (iii) 
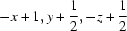
.

**Fig. 1.**
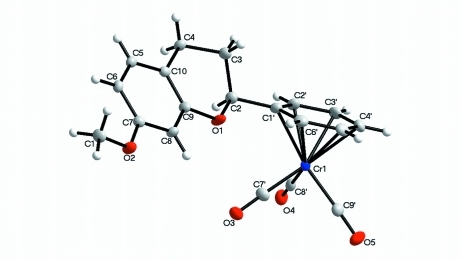
Diamond representation of the title compound, showing the numbering scheme and displacement ellipsoids (50% probability).

## supplementary crystallographic information

### Comment

The title compound, [Cr(CO)_3_(C_16_H_16_O_2_)] forms part of a series of chromium(0) complexes of the type [Cr(flav)(CO)_3_] (flav = flavan, flavone or isoflavone ligand) (van Tonder *et al.*, 2009*a*,*b* and 2010*a*,*b*). Our interest in this type of chromium(0) complexes is partly due to regioselective organic syntesis (Muschalek *et al.*, 2007) and to the general biological activity of flavanoids (Rice-Evans & Packer, 2003).

The title compound crystalized in the monoclinic space group P2(1)/c, with *Z* = 4. For the title compound the molecular structure displays the Cr(CO)_3_ moiety complexed to the flavone C-ring, exhibiting the known three-legged piano-stool conformation. All bond distances and angles are within range (Allen *et al.*, 1987). The distance between the Cr^0^ atom and the centroid of the A-η^6^-coordinated phenyl ring is 1.7119 (3) Å. The plane through C1'-C6' (r.m.s =0.0038 fitted atoms C1'-C6') of the phenyl ring is at an angle of 42.762 (43) ° to the plane formed between C4—C10 and O1 (r.m.s = 0.00824 fitted atoms C4, C5, C6, C7, C8, C9, C10 and O1). The dihydropyran ring displays a distored envelope configuration by displacement of atoms C2 and C3 from the fused ring system plane, with distances of 0.105 (2) 0.618 (2) Å respectively. The methoxy group at the C7 position is nearley coplanar to the plane created by the fused ring system. The methoxy forme a dihedral angle of -8.464 (23) ° between C1—O2—C7—C6. The molecules form chains through intermolecular C4—H4···O2^i^, C2—H2···O4^ii^ and C1—H1C···O4^iii^ hydrogen interactions (Table 1).

### Experimental

7-methoxyflavan-4-one was synthesized as described by Sato *et al.* (2006). 7-Methoxyflavan-4-one (1.00 g; 3.9 mmol), 10% Pd/C (0.10 g), 3 *M* H_2_SO_4_ (aq) (1 ml), ethanol (30 ml). Purification by means of flash column-chromatography yielded 7-methoxyflavan (0.67 g; 70.6%) as a colourless oil as described by Sato *et al.* (2006) *R_f_* 0.65 (hexane:DCM:ethyl acetate; 50:50:1); ^1^H NMR (600 MHz, CDCl_3_) δ 7.44–7.41 (m, H-2' and H-6', 2H), 7.40–7.37 (m, H-3' and H-5', 2H), 7.34–7.31 (m, H-4', 1H), 6.99–6.97 (m, H-5, 1H), 6.50–6.47 (m, H-6 and H-8, 2H), 5.05 (dd, *J* = 10.19, 2.37 Hz, H-2 1H), 3.77 (s,-OCH_3_, 3H), 2.92 (ddd, *J* = 16.08, 10.92, 6.02 Hz, H-4(*a*) 1H), 2.74 (ddd, *J* = 16.08, 5.12, 3.40 Hz, H-4(*e*) 1H), 2.22–2.18 (m, H-3 1H), 2.11–2.04 (m, H-3 1H); ^13^C NMR (151 MHz, CDCl_3_) δ p.p.m. 24.47 (C-4), 30.19 (C-3), 55.38 (–OCH_3_), 77.98 (C-2),101.71 (C-6/8), 107.54 (C-6/8), 114.01, 126.11, 127.93, 128.61, 130.05, 141.79, 155.91, 159.23.

Preparation of the title compound was based on a method described by Müller *et al.* (1999). A solution of 7-methoxyflavan (0.27 g, 1.1 mmol) and Cr(CO)_6_ (0.25 g, 1.1 mmol, 1 eq) in butylether:THF (9:1; 25 ml) was degassed with argon, using standard Schlenk techniques, and refluxed (70 hr) under an oxygen free atmosphere. The reaction mixture was cooled to room temperature and evaporated *in vacuo*. Purification through flash column-chromotography yielded the title compound (0.10 g; 24%) as a yellow solid. Recrystallization from hexane:dichloromethane (6:1) yielded yellow plate like crystals suitable for X-ray analysis.

*R_f_* 0.30 (hexane:ethyl acetate; 3:5); Mp 145–147 °C IR ν(CO) = 1952, 1894 and 1844 cm^-1^;^1^H NMR (600 MHz, CDCl_3_) δ 6.96 (d, *J* = 8.3 Hz, 1H), 6.49 (dd, *J* = 8.3, 2.5 Hz, 1H), 6.48 (d, *J* = 2.5 Hz, 1H), 5.56 (d, *J* = 6.2 Hz, 1H), 5.43–5.41 (m, 1H), 5.37–5.32 (m, 3H), 4.67 (dd, *J* = 10.4, 2.3 Hz, 1H), 3.77 (s, 3H), 2.94–2.87 (m, 1H), 2.76 (m, 1H), 2.25 (m, 1H), 1.99 (m, 1H). ^13^C NMR (151 MHz, CDCl_3_) δ p.p.m. 232.53, 159.27, 155.02, 129.85, 113.31, 111.38, 108.09, 101.51, 92.22, 91.62, 91.49, 91.22, 90.68, 75.37, 55.37, 29.89, 24.11.

### Refinement

All H atoms were positioned geometrically and allowed to ride on their parent atoms, with *U*_iso_(H) = 1.2*U*_eq_(parent) of the parent atom with a C—H distance of 0.93. The methine H atoms were placed in geometrically idealized positions and constrained to ride on its parent atoms with *U*_iso_(H) = 1.5*U*_eq_(C) and at a distance of 0.97 Å. The methyl H atoms were placed in geometrically idealized positions and constrained to ride on its parent atoms with *U*_iso_(H) = 1.5*U*_eq_(C) and at a distance of 0.96 Å.

### Crystal data

**Table:**

[Cr(C_16_H_16_O_2_)(CO)_3_]	*F*(000) = 776
*M**_r_* = 376.32	*D*_x_ = 1.528 Mg m^−^^3^
Monoclinic, *P*2_1_/*c*	Mo *K*α radiation, λ = 0.71073 Å
*a* = 9.7703 (5) Å	Cell parameters from 9920 reflections
*b* = 19.1820 (9) Å	θ = 3.0–28.2°
*c* = 8.8049 (4) Å	µ = 0.73 mm^−^^1^
β = 97.494 (2)°	*T* = 100 K
*V* = 1636.07 (14) Å^3^	Plate, yellow
*Z* = 4	0.34 × 0.23 × 0.08 mm

### Data collection

**Table:**

Bruker X8 APEXII 4K KappaCCD diffractometer	4069 independent reflections
graphite	3526 reflections with *I* > 2σ(*I*)
Detector resolution: 8.5 pixels mm^-1^	*R*_int_ = 0.033
φ and ω scans	θ_max_ = 28.3°, θ_min_ = 3.1°
Absorption correction: multi-scan (*SADABS*; Bruker, 2008)	*h* = −13→9
*T*_min_ = 0.818, *T*_max_ = 0.942	*k* = −25→25
29057 measured reflections	*l* = −11→11

### Refinement

**Table:**

Refinement on *F*^2^	Primary atom site location: structure-invariant direct methods
Least-squares matrix: full	Secondary atom site location: difference Fourier map
*R*[*F*^2^ > 2σ(*F*^2^)] = 0.034	Hydrogen site location: inferred from neighbouring sites
*wR*(*F*^2^) = 0.090	H-atom parameters constrained
*S* = 1.00	*w* = 1/[σ^2^(*F*_o_^2^) + (0.0434*P*)^2^ + 1.6967*P*] where *P* = (*F*_o_^2^ + 2*F*_c_^2^)/3
4069 reflections	(Δ/σ)_max_ = 0.015
222 parameters	Δρ_max_ = 0.73 e Å^−^^3^
0 restraints	Δρ_min_ = −0.52 e Å^−^^3^

### Special details

**Table:**

Experimental. The intensity data was collected on a Bruker X8 ApexII 4 K Kappa CCD diffractometer using an exposure time of 15 s/frame. A total of 1821 frames were collected with a frame width of 0.5° covering up to θ = 28.18° with 99.7% completeness accomplished.
Geometry. All s.u.'s (except the s.u. in the dihedral angle between two l.s. planes) are estimated using the full covariance matrix. The cell s.u.'s are taken into account individually in the estimation of s.u.'s in distances, angles and torsion angles; correlations between s.u.'s in cell parameters are only used when they are defined by crystal symmetry. An approximate (isotropic) treatment of cell s.u.'s is used for estimating s.u.'s involving l.s. planes.
Refinement. Refinement of *F*^2^ against ALL reflections. The weighted *R*-factor *wR* and goodness of fit *S* are based on *F*^2^, conventional *R*-factors *R* are based on *F*, with *F* set to zero for negative *F*^2^. The threshold expression of *F*^2^ > 2σ(*F*^2^) is used only for calculating *R*-factors(gt) *etc*. and is not relevant to the choice of reflections for refinement. *R*-factors based on *F*^2^ are statistically about twice as large as those based on *F*, and *R*- factors based on ALL data will be even larger.

### Fractional atomic coordinates and isotropic or equivalent isotropic displacement parameters (Å^2^)

**Table:**

	*x*	*y*	*z*	*U*_iso_*/*U*_eq_	
C1	0.3380 (2)	0.29331 (9)	0.6478 (2)	0.0208 (4)	
H1A	0.291	0.2854	0.7355	0.031*	
H1B	0.3066	0.3363	0.5995	0.031*	
H1C	0.4356	0.2959	0.6798	0.031*	
C1'	0.17467 (19)	−0.11113 (9)	0.36846 (19)	0.0178 (3)	
C2	0.28858 (19)	−0.07158 (9)	0.4658 (2)	0.0190 (3)	
H2	0.3777	−0.0837	0.4332	0.023*	
C2'	0.19981 (19)	−0.17979 (9)	0.3254 (2)	0.0196 (3)	
H2'	0.2859	−0.1997	0.355	0.023*	
C3'	0.0952 (2)	−0.21918 (9)	0.2369 (2)	0.0213 (4)	
H3'	0.1123	−0.265	0.2106	0.026*	
C3	0.29216 (19)	−0.08786 (9)	0.6350 (2)	0.0202 (4)	
H3A	0.3106	−0.1371	0.6528	0.024*	
H3B	0.2034	−0.0771	0.6676	0.024*	
C4'	−0.03356 (19)	−0.18914 (10)	0.1893 (2)	0.0219 (4)	
H4'	−0.1018	−0.2147	0.1301	0.026*	
C4	0.40480 (18)	−0.04451 (8)	0.72666 (19)	0.0167 (3)	
H4A	0.4003	−0.0501	0.8354	0.02*	
H4B	0.4948	−0.0602	0.7056	0.02*	
C5	0.43124 (17)	0.08604 (9)	0.77979 (19)	0.0159 (3)	
H5	0.4782	0.0757	0.8759	0.019*	
C5'	−0.05972 (19)	−0.11962 (10)	0.2316 (2)	0.0220 (4)	
H5'	−0.1454	−0.0995	0.2003	0.026*	
C6	0.41013 (17)	0.15570 (9)	0.73907 (19)	0.0159 (3)	
H6	0.4428	0.1912	0.8062	0.019*	
C6'	0.04282 (19)	−0.08107 (9)	0.3203 (2)	0.0197 (3)	
H6'	0.0248	−0.0355	0.348	0.024*	
C7	0.33885 (17)	0.17112 (9)	0.59536 (19)	0.0159 (3)	
C7'	0.20844 (19)	−0.03456 (9)	0.0866 (2)	0.0210 (2)	
C8	0.29047 (18)	0.11775 (9)	0.49617 (19)	0.0176 (3)	
H8	0.2422	0.1281	0.4007	0.021*	
C8'	0.26596 (17)	−0.16022 (8)	0.02184 (19)	0.0168 (3)	
C9	0.31439 (18)	0.04906 (9)	0.53999 (19)	0.0163 (3)	
C9'	0.02989 (18)	−0.11148 (9)	−0.0765 (2)	0.0185 (3)	
C10	0.38501 (17)	0.03117 (8)	0.68272 (19)	0.0148 (3)	
O1	0.25988 (15)	0.00078 (6)	0.43410 (14)	0.0232 (3)	
O2	0.30939 (14)	0.23734 (6)	0.54184 (14)	0.0210 (3)	
O3	0.26044 (13)	0.01758 (6)	0.06402 (15)	0.0210 (2)	
O4	0.35317 (13)	−0.18513 (7)	−0.03763 (15)	0.0218 (3)	
O5	−0.02741 (14)	−0.10672 (8)	−0.19953 (15)	0.0280 (3)	
Cr1	0.12859 (3)	−0.120499 (13)	0.11540 (3)	0.01295 (8)	

### Atomic displacement parameters (Å^2^)

**Table:**

	*U*^11^	*U*^22^	*U*^33^	*U*^12^	*U*^13^	*U*^23^
C1	0.0271 (9)	0.0133 (8)	0.0223 (9)	−0.0016 (6)	0.0046 (7)	−0.0035 (6)
C1'	0.0238 (9)	0.0148 (8)	0.0143 (8)	−0.0025 (6)	0.0010 (6)	0.0018 (6)
C2	0.0226 (8)	0.0155 (8)	0.0185 (8)	−0.0028 (6)	0.0008 (7)	0.0002 (6)
C2'	0.0249 (9)	0.0141 (8)	0.0184 (8)	−0.0011 (6)	−0.0022 (7)	0.0037 (6)
C3'	0.0295 (10)	0.0125 (7)	0.0214 (9)	−0.0055 (7)	0.0017 (7)	0.0026 (6)
C3	0.0240 (9)	0.0170 (8)	0.0189 (8)	−0.0025 (7)	0.0007 (7)	0.0026 (6)
C4'	0.0204 (9)	0.0238 (9)	0.0215 (8)	−0.0092 (7)	0.0030 (7)	0.0029 (7)
C4	0.0190 (8)	0.0152 (8)	0.0149 (7)	0.0005 (6)	−0.0017 (6)	0.0014 (6)
C5	0.0134 (7)	0.0194 (8)	0.0145 (7)	−0.0003 (6)	−0.0003 (6)	0.0010 (6)
C5'	0.0157 (8)	0.0282 (9)	0.0231 (9)	−0.0005 (7)	0.0062 (7)	0.0040 (7)
C6	0.0146 (8)	0.0168 (8)	0.0160 (8)	−0.0024 (6)	0.0013 (6)	−0.0034 (6)
C6'	0.0243 (9)	0.0188 (8)	0.0170 (8)	0.0011 (7)	0.0072 (7)	−0.0003 (6)
C7	0.0168 (8)	0.0143 (7)	0.0169 (8)	0.0001 (6)	0.0029 (6)	0.0005 (6)
C7'	0.0240 (5)	0.0159 (4)	0.0236 (5)	−0.0015 (4)	0.0056 (4)	0.0004 (4)
C8	0.0218 (8)	0.0169 (8)	0.0130 (7)	−0.0012 (6)	−0.0016 (6)	0.0016 (6)
C8'	0.0179 (8)	0.0128 (7)	0.0184 (8)	−0.0028 (6)	−0.0019 (6)	−0.0013 (6)
C9	0.0215 (8)	0.0140 (7)	0.0134 (7)	−0.0029 (6)	0.0019 (6)	−0.0017 (6)
C9'	0.0143 (8)	0.0202 (8)	0.0213 (8)	0.0005 (6)	0.0036 (6)	−0.0005 (6)
C10	0.0134 (7)	0.0159 (7)	0.0151 (7)	0.0004 (6)	0.0025 (6)	0.0016 (6)
O1	0.0419 (8)	0.0117 (6)	0.0135 (6)	−0.0046 (5)	−0.0057 (5)	0.0009 (4)
O2	0.0314 (7)	0.0122 (6)	0.0177 (6)	0.0000 (5)	−0.0027 (5)	0.0002 (5)
O3	0.0240 (5)	0.0159 (4)	0.0236 (5)	−0.0015 (4)	0.0056 (4)	0.0004 (4)
O4	0.0190 (6)	0.0206 (6)	0.0258 (7)	0.0003 (5)	0.0032 (5)	−0.0063 (5)
O5	0.0236 (7)	0.0394 (8)	0.0195 (7)	0.0032 (6)	−0.0032 (5)	0.0001 (6)
Cr1	0.01308 (14)	0.01116 (13)	0.01423 (14)	−0.00064 (9)	0.00033 (9)	−0.00028 (9)

### Geometric parameters (Å, °)

**Table:**

C1—O2	1.426 (2)	C4—H4A	0.97
C1—H1A	0.96	C4—H4B	0.97
C1—H1B	0.96	C5—C6	1.392 (2)
C1—H1C	0.96	C5—C10	1.393 (2)
C1'—C2'	1.401 (2)	C5—H5	0.93
C1'—C6'	1.424 (3)	C5'—C6'	1.399 (3)
C1'—C2	1.517 (2)	C5'—Cr1	2.2187 (18)
C1'—Cr1	2.2221 (17)	C5'—H5'	0.93
C2—O1	1.436 (2)	C6—C7	1.394 (2)
C2—C3	1.518 (2)	C6—H6	0.93
C2—H2	0.98	C6'—Cr1	2.2188 (17)
C2'—C3'	1.419 (2)	C6'—H6'	0.93
C2'—Cr1	2.2044 (17)	C7—O2	1.372 (2)
C2'—H2'	0.93	C7—C8	1.388 (2)
C3'—C4'	1.397 (3)	C7'—O3	1.151 (2)
C3'—Cr1	2.2194 (17)	C7'—Cr1	1.8552 (18)
C3'—H3'	0.93	C8—C9	1.384 (2)
C3—C4	1.524 (2)	C8—H8	0.93
C3—H3A	0.97	C8'—O4	1.160 (2)
C3—H3B	0.97	C8'—Cr1	1.8300 (18)
C4'—C5'	1.417 (3)	C9—O1	1.3716 (19)
C4'—Cr1	2.2216 (17)	C9—C10	1.395 (2)
C4'—H4'	0.93	C9'—O5	1.156 (2)
C4—C10	1.508 (2)	C9'—Cr1	1.8413 (18)
			
O2—C1—H1A	109.5	C5'—C6'—C1'	120.42 (16)
O2—C1—H1B	109.5	C5'—C6'—Cr1	71.62 (10)
H1A—C1—H1B	109.5	C1'—C6'—Cr1	71.42 (10)
O2—C1—H1C	109.5	C5'—C6'—H6'	119.8
H1A—C1—H1C	109.5	C1'—C6'—H6'	119.8
H1B—C1—H1C	109.5	Cr1—C6'—H6'	129.6
C2'—C1'—C6'	118.91 (16)	O2—C7—C8	115.32 (14)
C2'—C1'—C2	118.79 (16)	O2—C7—C6	124.44 (15)
C6'—C1'—C2	122.29 (15)	C8—C7—C6	120.24 (15)
C2'—C1'—Cr1	70.86 (10)	O3—C7'—Cr1	177.22 (16)
C6'—C1'—Cr1	71.16 (10)	C9—C8—C7	119.69 (15)
C2—C1'—Cr1	130.16 (12)	C9—C8—H8	120.2
O1—C2—C1'	105.27 (14)	C7—C8—H8	120.2
O1—C2—C3	111.62 (14)	O4—C8'—Cr1	179.72 (16)
C1'—C2—C3	111.90 (14)	O1—C9—C8	114.63 (14)
O1—C2—H2	109.3	O1—C9—C10	123.27 (15)
C1'—C2—H2	109.3	C8—C9—C10	122.07 (15)
C3—C2—H2	109.3	O5—C9'—Cr1	177.22 (16)
C1'—C2'—C3'	120.66 (17)	C5—C10—C9	116.69 (15)
C1'—C2'—Cr1	72.24 (10)	C5—C10—C4	123.31 (15)
C3'—C2'—Cr1	71.86 (10)	C9—C10—C4	119.97 (15)
C1'—C2'—H2'	119.7	C9—O1—C2	118.15 (13)
C3'—C2'—H2'	119.7	C7—O2—C1	117.25 (13)
Cr1—C2'—H2'	128.5	C8'—Cr1—C9'	87.31 (8)
C4'—C3'—C2'	120.04 (17)	C8'—Cr1—C7'	87.84 (8)
C4'—C3'—Cr1	71.75 (10)	C9'—Cr1—C7'	88.31 (8)
C2'—C3'—Cr1	70.71 (10)	C8'—Cr1—C2'	89.59 (7)
C4'—C3'—H3'	120	C9'—Cr1—C2'	152.67 (7)
C2'—C3'—H3'	120	C7'—Cr1—C2'	118.72 (7)
Cr1—C3'—H3'	130.1	C8'—Cr1—C5'	155.74 (7)
C2—C3—C4	109.27 (14)	C9'—Cr1—C5'	93.20 (7)
C2—C3—H3A	109.8	C7'—Cr1—C5'	116.42 (8)
C4—C3—H3A	109.8	C2'—Cr1—C5'	78.89 (7)
C2—C3—H3B	109.8	C8'—Cr1—C6'	152.24 (7)
C4—C3—H3B	109.8	C9'—Cr1—C6'	120.40 (7)
H3A—C3—H3B	108.3	C7'—Cr1—C6'	91.05 (7)
C3'—C4'—C5'	119.71 (16)	C2'—Cr1—C6'	66.76 (7)
C3'—C4'—Cr1	71.57 (10)	C5'—Cr1—C6'	36.75 (7)
C5'—C4'—Cr1	71.28 (10)	C8'—Cr1—C3'	91.40 (7)
C3'—C4'—H4'	120.1	C9'—Cr1—C3'	115.48 (7)
C5'—C4'—H4'	120.1	C7'—Cr1—C3'	156.15 (8)
Cr1—C4'—H4'	129.4	C2'—Cr1—C3'	37.43 (6)
C10—C4—C3	109.26 (13)	C5'—Cr1—C3'	66.50 (7)
C10—C4—H4A	109.8	C6'—Cr1—C3'	78.69 (7)
C3—C4—H4A	109.8	C8'—Cr1—C1'	114.87 (7)
C10—C4—H4B	109.8	C9'—Cr1—C1'	157.81 (7)
C3—C4—H4B	109.8	C7'—Cr1—C1'	91.79 (7)
H4A—C4—H4B	108.3	C2'—Cr1—C1'	36.90 (6)
C6—C5—C10	122.81 (15)	C5'—Cr1—C1'	66.97 (7)
C6—C5—H5	118.6	C6'—Cr1—C1'	37.42 (7)
C10—C5—H5	118.6	C3'—Cr1—C1'	66.98 (6)
C6'—C5'—C4'	120.25 (17)	C8'—Cr1—C4'	118.55 (7)
C6'—C5'—Cr1	71.63 (10)	C9'—Cr1—C4'	90.93 (7)
C4'—C5'—Cr1	71.51 (10)	C7'—Cr1—C4'	153.54 (8)
C6'—C5'—H5'	119.9	C2'—Cr1—C4'	66.91 (7)
C4'—C5'—H5'	119.9	C5'—Cr1—C4'	37.21 (7)
Cr1—C5'—H5'	129.4	C6'—Cr1—C4'	66.71 (7)
C5—C6—C7	118.50 (15)	C3'—Cr1—C4'	36.68 (7)
C5—C6—H6	120.8	C1'—Cr1—C4'	79.24 (7)
C7—C6—H6	120.8		

### Hydrogen-bond geometry (Å, °)

**Table:**

*D*—H···*A*	*D*—H	H···*A*	*D*···*A*	*D*—H···*A*
C4'—H4'···O2^i^	0.93	2.54	3.459 (2)	169
C2'—H2'···O4^ii^	0.93	2.46	3.153 (2)	132
C1—H1C···O4^iii^	0.96	2.57	3.314 (2)	134

Symmetry codes: (i) −*x*, *y*−1/2, −*z*+1/2; (ii) *x*, −*y*−1/2, *z*+1/2; (iii) −*x*+1, *y*+1/2, −*z*+1/2.
